# Recurrence of Graves’ Disease: What Genetics of HLA and *PTPN22* Can Tell Us

**DOI:** 10.3389/fendo.2021.761077

**Published:** 2021-11-23

**Authors:** Daniela Vejrazkova, Josef Vcelak, Eliska Vaclavikova, Marketa Vankova, Katerina Zajickova, Jana Vrbikova, Michaela Duskova, Petra Pacesova, Zdenek Novak, Bela Bendlova

**Affiliations:** ^1^ Department of Molecular Endocrinology, Institute of Endocrinology, Prague, Czechia; ^2^ Department of Clinical Endocrinology, Institute of Endocrinology, Prague, Czechia; ^3^ Department of Steroids and Proteohormones, Institute of Endocrinology, Prague, Czechia

**Keywords:** Graves’ disease, HLA variants, *PTPN22* gene, genetic predictors, treatment

## Abstract

**Background:**

Approximately half of patients diagnosed with Graves’ disease (GD) relapse within two years of thyreostatic drug withdrawal. It is then necessary to decide whether to reintroduce conservative treatment that can have serious side effects, or to choose a radical approach. Familial forms of GD indicate a significant genetic component. Our aim was to evaluate the practical benefits of HLA and *PTPN22* genetic testing for the assessment of disease recurrence risk in the Czech population.

**Methods:**

In 206 patients with GD, exon 2 in the HLA genes *DRB1*, *DQA1*, *DQB1* and rs2476601 in the gene *PTPN22* were sequenced.

**Results:**

The risk HLA haplotype DRB1*03-DQA1*05-DQB1*02 was more frequent in our GD patients than in the general European population. During long-term retrospective follow-up (many-year to lifelong perspective), 87 patients relapsed and 26 achieved remission lasting over 2 years indicating a 23% success rate for conservative treatment of the disease. In 93 people, the success of conservative treatment could not be evaluated (thyroidectomy immediately after the first attack or ongoing antithyroid therapy). Of the examined genes, the HLA-DQA1*05 variant reached statistical significance in terms of the ability to predict relapse (p=0.03). Combinations with either both other HLA risk genes forming the risk haplotype DRB1*03-DQA1*05-DQB1*02 or with the *PTPN22* SNP did not improve the predictive value.

**Conclusion:**

the DQA1*05 variant may be a useful prognostic marker in patients with an unclear choice of treatment strategy.

## Introduction

Graves’ disease (GD) is the most common cause of hyperthyroidism, affecting approximately 0.5% of men and 3% of women. In Europe, the first choice treatment is most often the administration of antithyroid drugs (1, 2). However, approximately half of patients relapse within two years of drug withdrawal. It is then necessary to decide whether to reintroduce thyreostatic therapy that may have serious side effects, or to choose a radical approach - total thyroidectomy (TTE) or radioiodine treatment.

A significant genetic component is evident from the familial occurrence of the disease. Studies on twin pairs have shown that the contribution of genetic factors can be as high as 70-80% (3). The autoimmune nature of GD has been associated with the human leukocyte antigen complex (HLA) as well as with the gene *PTPN22* (protein tyrosine phosphatase, nonreceptor type 22) on chromosome 1p13.3-13.1 encoding for protein tyrosine phosphatase-22, a powerful inhibitor of T-cell activation. Within the HLA, DRB1*03, DQA1*05, and DQB1*02 allelic groups have proven to be promising predictors of the development and recurrence of GD in some studies (4). Outside the HLA region, the *PTPN22* genetic variant rs2476601 has also been shown to be a potential Graves’ disease predictor and together with the HLA variants was included in the Graves’ Events After Therapy + (GREAT+) score (5). When individuals with a sensitive genetic background are exposed to certain environmental risk factors such as stress (6–8), smoking (9), iodine overdose (10), the postpartum period in women (11), microbiome-associated immunological changes (12) and possibly their combination, the production of autoantigens against the TSH receptor is triggered and the disease begins to develop or relapse. In addition to hyperthyroidism, extrathyroidal manifestations like Graves’ orbitopathy, thyroid dermatopathy, and rarely acropachy may be present. The long-term conservative treatment of persistent and recurrent hyperthyroidism entails considerable medical expenses and may have serious side effects in some cases, notably liver disorders and agranulocytosis.

In this retrospective study, we followed up on a pilot study published three years ago (13) with the aim to evaluate practical benefits of HLA and *PTPN22* genetic testing for assessments of the disease recurrence risk in the Czech population. Taking genetics into account as part of long-term follow-up would make it easier for physicians and patients to consider the suitability and optimal timing of radical and definitive approaches to treatment.

## Materials and Methods

### Participants

We analyzed the HLA-*DRB1*, HLA-*DQA1* and HLA-*DQB1* allelic groups as well as the *PTPN22* polymorphism rs2476601 (referred to also as 1858C/T) in 206 retrospectively observed Czech patients who had been diagnosed with Graves’ disease according to hormonal profile [low/suppressed TSH with simultaneously elevated freeT4, freeT3, presence of thyrotropin receptor antibodies (TRAK)] as well as sonographic examination of the thyroid gland. Orbit ultrasound had been performed in patients with present and active thyroid eye disease as a complementary exam while considering corticosteroids. Patients were recruited during the years 2017-2020 mainly from the outpatients of the Institute of Endocrinology in Prague. These patients are continuously monitored at the Institute, which gives us detailed long-term or even lifelong retrospective information on the status of their remission/relapse. An additional 16 patients were recruited from the outpatient Clinic of Endocrinology and Diabetology, M-centrum, Chotěboř, 3 patients were treated in the outpatient Clinic of Endocrinology in Říčany, and 3 patients were from the 3^rd^ Medical Department, 1^st^ Faculty of Medicine, Charles University and General Faculty Hospital in Prague. All participants gave their informed consent for inclusion before they participated in the study. The study was conducted in accordance with the Declaration of Helsinki, and the protocol was approved by the Ethics Committee of the Institute of Endocrinology (MH CR-RVO 00023761, date of approval 26. 6. 2017).

There were 171 women and 35 men in our cohort, and the mean age of patients at the time of diagnosis was 42.0 ± 14.54 years with median being 41 years and the age range spanning from 16 to 78 years. The mean duration of thyreostatic administration was 36.3 ± 42.47 months. Methimazol, or in case of intolerance propylthiouracyl, were prescribed. Systemic treatment of orbitopathy by peroral prednisone and/or by intravenous methylprednisolone pulses was indicated in 22.9% of the patients. Details regarding sex differences at the time of diagnosis, or in chronically ill patients at the time of the last relapse of GD, as well as retrospective specification of treatment are given in **Table 1**.

**Table 1:** Clinical characterization of the whole cohort of GD patients.

Characterization before treatment	Women (N = 171)	Men (N = 35)	p-level
Age (years)	42.0 ± 14.44	43.9 ± 15.20	0.71*
Volume of goiter (ml)	20.9 ± 12.10	29.1 ± 17.31	0.01*
TSH (mIU/l) - normal range 0.27 to 4.2	0.018 ± 0.0977	0.011 ± 0.0153	0.62*
fT4 (pmol/l) - normal range 12 to 22	48.4 ± 23.09	46.9 ± 24.84	0.66*
fT3 (pmol/l) - normal range 3.1 to 6.8	18.5 ± 12.70	21.2 ± 13.86	0.39*
**Way of treatment**			
Thyreostatic administration (months)	37.8 ± 44.79	27.9 ± 25.54	0.28*
Systemic treatment of orbitopathy (%)	23.9	18.2	0.47†
Thyroid surgery (%)	36.8	36.7	0.99†
RI administration (%)	10.43	13.8	0.58†

Data are given as mean ± SD or as a percentage; p-level according to *Mann-Whitney test or according to †Chi-square test.

Statistically significant p-levels are in bold.

### Genetic Analysis

Next generation sequencing performed on a MiSeq (Illumina) was used for haplotyping. In the three HLA genes (*DRB1*, *DQA1* and *DQB1*), exon 2 was amplified, since this part of the HLA molecule determines its antigenic properties. Amplification of DNA sequences was performed using certified kits (CE 0197, CE IVD) of allele-specific pre-coated PCR strips PROTRANS S4 HLA-DRB1 * (cat. No. 34 04), PROTRANS S3 HLA-DQB1 * (cat. No. 33 09) and PROTRANS S2 HLA-DQA1 * (cat. No. 32 08) from the manufacturer PentaGen, Protrans, Germany. A public IPD-IMGT/HLA (International ImMunoGeneTics Information System/HLA database) alignment tool (https://www.ebi.ac.uk/ipd/imgt/hla/align.html) (14) was used to identify the allelic groups after sequencing. For the DRB1 and DQB1 HLA allelic groups, the Allele Frequencies website (http://www.allelefrequencies.net/) (15) allowed a comparison of the obtained allele frequencies of GD patients with the Czech bone marrow donors (n=5099).

The polymorphism rs2476601; c.1858C> T p. (Arg620Trp) was analyzed by sequencing exon 14 of the *PTPN22* gene (protein tyrosine phosphatase, nonreceptor type 22) by next generation sequencing on a MiSeq sequencer (Illumina) using the Nextera XT kit (Illumina) to prepare libraries.

### Biochemical Analysis

Serum TSH, fT4 and fT3 concentrations were measured using the ECLIA method (Cobas Integra, Roche Diagnostics). For detection of autoantibodies to the TSH receptor, a fully automated electrochemiluminescence immunoassay Elecsys Anti-TSHR (TRAK) was used (Elecsys 2010, Cobas e601, Roche). The precision of this method is less than 6%, sensitivity 97% and specificity 99,5%.

### Statistical Analysis

Statistical analyses were performed using the NCSS/Pass 2004 software (NCSS, LLC, Kaysville, Utah, USA). Data are presented as means ± SD or as a percentage. The Chi-square test was used to compare the distribution of allelic groups in the particular cohorts. Odds ratios, relative risk and 95% confidence intervals were calculated in MedCalc Software. Differences in anamnestic data between men and women were tested by the non-parametric Mann-Whitney test. All tests were two-tailed (both positive and negative differences were considered). The p-value<0.05 threshold was used to suggest statistical significant differences.

## Results

### Evaluation of the Success of Conservative Treatment

As regards the distinction between successfully and unsuccessfully treated patients, we followed these criteria: patients remaining in remission at least 2 years since discontinuation of thyreostatic treatment and with no relapse throughout life were considered successfully treated. On the contrary, those who had relapsed at least once during monitoring at our institution (many-year to lifelong perspective) as well as patients requiring thyreostatics for more than 5 years were considered unsuccessfully treated. Of the 206 patients examined, 87 met the criteria for unsuccessful treatment, 26 patients met the criteria for successful treatment, indicating a 23% success rate for conservative treatment of the disease in long term follow-up. In 93 people, the success of treatment could not be evaluated because they either underwent TTE immediately after the first attack (so it was not possible to determine whether the disease would relapse), they are still on antithyroid therapy (for a period of less than 5 years, so they cannot yet be classified as unsuccessfully treated), or their remission has not yet reached two years (so they cannot yet be classified as successfully treated).

Comparison of patients with successful and unsuccessful conservative treatment is given in **Table 2**. Significant difference was observed in fT3 levels. Unsuccessfully treated patients showed twice as high concentrations compared to patients in remission. For fT4 levels, such differences between the two groups were not apparent.

**Table 2:** Comparison of patients with successful and unsuccessful conservative treatment.

Characterization before treatment	Successful treatment (N = 26)	Unsuccessful treatment (N = 87)	p-level
Age (years)	41.0 ± 13.05	44.9 ± 14.23	0.17*
Volume of goiter (ml)	17.8 ± 7.82	22.0 ± 13.43	0.27*
TSH (mIU/l) - normal range 0.27 to 4.2	0.011 ± 0.0160	0.013 ± 0.0198	0.80*
fT4 (pmol/l) - normal range 12 to 22	43.6 ± 19.12	46.6 ± 24.27	1.00*
fT3 (pmol/l) - normal range 3.1 to 6.8	10.4 ± 4.78	21.5 ± 15.37	0.03*
**Way of treatment**			
Thyreostatic administration (months)	25.1 ± 17.02	56.1 ± 55.11	0.002*
Systemic treatment of orbitopathy (%)	9.1	24.1	12.4†
Thyroid surgery (%)	0	31.7	–
RI administration (%)	0	12.0	–

Data are given as mean ± SD or as a percentage; p-level according to *Mann-Whitney test or according to †Chi-square test.

Statistically significant p-levels are in bold.

### Frequencies of the HLA Allelic Groups

The risk haplotype DRB1*03-DQA1*05-DQB1*02 was present in 35% of the entire cohort of 206 GD patients, 5 patients were homozygotes in this haplotype, 67 were heterozygotes. Haplotype frequency in the whole cohort was 18.7%. This highly exceeds the frequencies in non-GD populations as given in the Allele Frequencies website. For the individual HLA genes and the proportion of allelic groups in the whole cohort of GD patients, see **Table 3**.

**Table 3:** Frequencies of the HLA allelic groups in the whole cohort of GD patients, N = 206.

HLA genes	Allelic groups of individual HLA genes															
	*01	*02	*03	*04	*05	*06	*07	*08	*09	*10	*11	*12	*13	*14	*15	*16
***DRB1***	9	–	**21**	10	–	–	6	4	1	0.5	16	1.5	12	2	13	4
***DQA1***	37	6	11	3	**42**	1	–	–	–	–	–	–	–	–	–	–
***DQB1***	–	**26**	34	3	16	21	–	–	–	–	–	–	–	–	–	–

Data are given as %, frequencies of the risk variants of the particular genes are in bold.

The asterix represent an established form of HLA genes nomenclature in accordance with the current HLA nomenclature (http://hla.alleles.org/nomenclature/nomenc_updates.html).

For the frequencies of the HLA allelic groups analyzed separately in the unsuccessfully and successfully treated cohort as well as in the patients whose success of treatment could not be evaluated, see **Tables 4**–**6**, respectively.

**Table 4:** Frequencies of the HLA allelic groups in unsuccessfully treated GD patients, N = 87.

HLA genes	Allelic groups of individual HLA genes															
	*01	*02	*03	*04	*05	*06	*07	*08	*09	*10	*11	*12	*13	*14	*15	*16
***DRB1***	10	–	**23**	9	–	–	3	4	0.5	0.5	18	2	15	3	10	2
***DQA1***	35	3	10	3	**48**	1	–	–	–	–	–	–	–	–	–	–
***DQB1***	–	**25**	37	3	15	20	–	–	–	–	–	–	–	–	–	–

Data are given as %, frequencies of the risk variants of the particular genes are in bold.

The asterix represent an established form of HLA genes nomenclature in accordance with the current HLA nomenclature (http://hla.alleles.org/nomenclature/nomenc_updates.html).

**Table 5:** Frequencies of the HLA allelic groups in successfully treated GD patients, N = 26.

HLA genes	Allelic groups of individual HLA genes															
	*01	*02	*03	*04	*05	*06	*07	*08	*09	*10	*11	*12	*13	*14	*15	*16
***DRB1***	10	–	**18**	10	–	–	10	4	0	2	10	0	10	2	18	6
***DQA1***	46	10	10	4	**30**	0	–	–	–	–	–	–	–	–	–	–
***DQB1***	–	**26**	22	6	20	26	–	–	–	–	–	–	–	–	–	–

Data are given as %, frequencies of the risk variants of the particular genes are in bold.

The asterix represent an established form of HLA genes nomenclature in accordance with the current HLA nomenclature (http://hla.alleles.org/nomenclature/nomenc_updates.html).

**Table 6:** Frequencies of the HLA allelic groups in GD patients whose success of treatment could not be evaluated, N = 93.

HLA genes	Allelic groups of individual HLA genes															
	*01	*02	*03	*04	*05	*06	*07	*08	*09	*10	*11	*12	*13	*14	*15	*16
***DRB1***	9	–	**19.5**	11	–	–	8	3	1	0	15.5	1.5	10.5	1	14	6
***DQA1***	36	8.5	12.5	2.5	**40**	0.5	–	–	–	–	–	–	–	–	–	–
***DQB1***	–	**26.5**	35	2.5	15.5	20.5	–	–	–	–	–	–	–	–	–	–

Data are given as %, frequencies of the risk variants of the particular genes are in bold.

The asterix represent an established form of HLA genes nomenclature in accordance with the current HLA nomenclature (http://hla.alleles.org/nomenclature/nomenc_updates.html).

Evaluated in the overall cohort of 206 participants, the allele DRB1*03 was more frequent in GD patients (21%) compared to the Czech bone marrow donors (12%) we used as a reference, see details in Methods. On the contrary, the DRB1*07 allele was less frequent in our GD group (6%) in comparison with bone marrow donors (14%), indicating its protective effect in terms of disease development. It is noteworthy that, in the unsuccessfully treated subgroup, the DRB1*07 allele frequency reached only 3%, while in the successfully treated subgroup it reached 10% (p=0.05), indicating a protective effect of the *07 variant even in terms of disease relapse. The risk variant DQA1*05 was present in 67.0% of our GD patients with an allele frequency of 42%, 17.5% of the patients (36 individuals) were homozygotes in this allelic group. The proportion of these homozygotes reached 18.1% in unsuccessfully treated patients, whereas it was only 12.0% in successfully treated patients (p=0.035). Unfortunately, for the HLA-*DQA1* gene, a comparison with the Czech bone marrow donors was not available; however, according to the Allele Frequencies website, the allele frequency observed in our study in Czech GD patients was markedly higher than in the other listed European populations, see the *Discussion* for details. The frequency of the risk variant DQB1*02 observed in our group of patients (26%) was comparable to the frequency in Czech bone marrow donors (23%). No significant differences in the distribution of the particular risk allelic groups DRB1*03, DQA1*05 and DQB1*02 or of the risk haplotype DRB1*03-DQA1*05-DQB1*02 were observed between men and women.

Complete allele, phenotype and haplotype frequencies of our cohort of 206 GD patients were published in public repository Figshare.com (16) with DOI for public link: 10.6084/m9.figshare.16446954. The raw sequencing data are available in the public repository NCBI (17), accession ID to cite for the Sequence Read Archive (SRA) data: PRJNA776790, public link to access the SRA records: https://www.ncbi.nlm.nih.gov/sra/PRJNA776790.

### Success of Conservative Treatment in Relation to Genotype

An evaluation of the three HLA genes and the *PTPN22* SNP in terms of their association with treatment success showed that only the HLA-DQA1*05 variant reached statistical significance (p=0.03), as detailed in **Table 7**:

**Table 7:** Distribution of the studied genetic variants in the group of GD patients depending on the success of the treatment, N = 113.

DRB1*03 presence	Successful conservative treatment		Statistics
	**no**	**yes**	Chi^2^-test=0.59
**no**	53	18	power=0.12
**yes**	34	8	p=0.44
DQB1*02 presence	**Successful conservative treatment**		**Statistics**
	**no**	**yes**	Chi^2^-test=0.0003
**no**	47	14	power=0.05
**yes**	40	12	p=0.99
DQA1*05 presence	**Successful conservative treatment**		**Statistics**
	**no**	**yes**	Chi^2^-test=4.69
**no**	21	12	power=0.6
**yes**	66	14	**p=0.03**
DRB1*03-DQB1*02-DQA1*05 haplotype	**Successful conservative treatment**		**Statistics**
	**no**	**yes**	Chi^2^-test=0.44
**no**	54	18	power=0.10
**yes**	33	8	p=0.51
***PTPN22* variant**	**Successful conservative treatment**		**Statistics**
	**no**	**yes**	Chi^2^-test=1.62
**no**	59	21	power=0.25
**yes**	28	5	p=0.20

Statistically significant p-levels are in bold.

The asterix represent an established form of HLA genes nomenclature in accordance with the current HLA nomenclature (http://hla.alleles.org/nomenclature/nomenc_updates.html).

There were 14 successfully treated patients among the 80 DQA1*05 carriers (17.5%), while there were 66 DQA1*05 carriers among the 87 unsuccessfully treated patients (75.9%). The combination of the DQA1*05 variant with both other risk HLA genetic variants forming the risk haplotype DRB1*03-DQA1*05-DQB1*02 and with the *PTPN22* SNP did not improve the ability to predict relapse. The odds ratio (OR) for relapse (or more precisely, for unsuccessful conservative treatment, that is, for relapse of the disease or for prolonged inconclusive treatment lasting more than 5 years, which we observed in several cases) calculated as the ratio of the odds of the unsuccessful treatment occurring in the group of risk allele DQA1*05 carriers to the odds of it occurring in the group of risk allele DQA1*05 non-carriers was 2.7 (95%CI: 1.08-6.72); p=0.05. The relative risk was 1.3 (95%CI: 0.98-1.71); p=0.07.

The DRB1*03 variant was not associated with treatment outcome (p=0.44), nor was the allelic group DQB1*02 (p=0.73), see **Table 7**.

As regards rs2476601 (1858C/T) in the *PTPN22* gene, the minor allele was present in 27% of patients, with an allelic frequency of 14.1%. The allelic frequency in unsuccessfully treated GD patients was 16.7%, in successfully treated patients 12.0% (Chi2-test=0.45; p=0.50). In patients with impossible evaluation of the treatment success, the allelic frequency was 12.4%. The OR calculated for relapse was 1.99, (95%CI: 0.68-5.83); p=0.30, and the relative risk was 1.15 (95%CI: 0.95-1.40); p=0.16. Although this polymorphism causing the substitution of arginine to tryptophan at codon 620 was found to be associated with GD in many European studies, its association with disease relapse was not apparent in our data.

## Discussion

Studies in the past two decades have clearly shown that multiple factors are involved in the development and recurrence of GD. The interaction of susceptible genes with variable environmental factors, mediated through very complex endogenous communication including dynamic epigenetic modulation and gene expression changes, may lead to impaired immunological tolerance and the outbreak of the disease (18–20). In addition to HLA variants, many non-HLA genes have been confirmed to be involved in the etiology of GD; some are unique to the recurrence (21, 22) of GD, while others are common to both the disease onset and recurrence, or pose a risk for the development of an even wider range of autoimmune diseases (23).

According to our data, the risk HLA haplotype DRB1*03-DQA1*05-DQB1*02 was more frequent in GD patients than in the general European population, as given in the Allele Frequencies website, with the two exceptions of Sardinia and Slovenia, that are represented by small groups (n ≤ 140). For the relatively small Czech control population sample in this website (n=180), a frequency of 9.2% is given for the haplotype DRB1*03-DQA1*05:01-DQB1*02:01. The haplotype frequency observed in our cohort of patients (18.7%) corresponds with a metaanalysis on GD patients by Gough (4). The risk allelic group DRB1*03 was more frequent in the GD group compared to the Czech bone marrow donors, which is in agreement with previous findings in other populations (24). On the other hand, the DRB1*07 allele was less frequent in our GD group in comparison with bone marrow donors and its frequency was clearly the lowest in unsuccessfully treated patients. The DRB1*07 allele has been reported to be protective for GD in UK Caucasians (25); however, due to its low incidence, a larger cohort of patients would be needed to demonstrate its significant protective role in the Czech population.

The allele frequency of the risk variant DQA1*05 was markedly higher in our study (42%) than in other European populations, ranging from 16.6% in France to 30.3% in Belgium according to the Allele Frequencies website. The DQA1*05 variant showed the best, though borderline, predictive ability in terms of disease relapse, with an OR=2.7. Although it cannot be considered a reliable recurrence predictor, its evaluation may be useful in patients with a complex clinical picture and unclear treatment strategy.

The tyrosine phosphatase-22 protein encoded by the *PTPN22* gene inhibits T-cell activation. The substitution of arginine to tryptophan at codon 620 (rs2476601) disrupts an interaction motif in the protein (26). This SNP has been associated with GD in many studies. These associations have shown high reproducibility in Caucasian populations, with an OR of 1.9 in British Caucasians (27), 1.7 in a Polish population (28), and even 4.2 in a Russian population (29); however, in Asian and African populations the minor allele was sporadic or absent (30). The concept of our study does not allow the calculation of OR in this sense, because we worked only with a group of patients and have no comparison with the Czech healthy controls for this SNP. However, the OR calculated for relapse did not show statistical significance. A comparison with some reference European populations suggests that there is higher minor allele frequency in our GD patients (14.1%). For example, the minor allele frequency in controls was reported as 10.4% in the United Kingdom (31), 10.0% in Germany (32) and 9% in Italy (33). However, the northernmost and easternmost countries of the continent show minor allele frequencies in the general population similar to our GD cohort (15% in Finland and 14.1% in the Ukraine (34). Either way, our study did not demonstrate the predictive potential of the *PTPN22* variant in terms of disease recurrence.

It is worth noting the observation of significantly higher fT3 values - but not fT4 values - in patients whose conservative treatment has not been successful for a long time. It is consistent with the conclusions of the original (35) and review articles (36, 37) on this topic, which describe a higher fT3 to fT4 ratio in patients who failed to respond to antithyroid drug therapy. This association may be mediated through variability in deiodinase type 2 gene, which affects activity of type 2 deiodinase regulating the transformation of T4 into T3 (38).

The absence of a control group of healthy Czech volunteers, ideally without history of autoimmune disorders, is one limitation for an interpretation of the data presented here. Furthermore, the size of the cohort suitable for assessing the predictive ability in terms of disease relapse should be larger, considering that only 113 people could be included in the statistical evaluation. On the other hand, an advantage of our study is the availability of medical history data and retrospective course of the disease of almost all patients in the sample, as they are persons receiving long-term and often life-long care in our institution. This approach indicates a 23% success rate for conservative treatment. Available medical history will also allow for a re-evaluation after several years, when it will be interesting to verify how many participants now belonging to the group of successfully treated patients in remission will move to the group of those who relapsed, and how these potential changes will affect the statistical results.

In conclusion, we analyzed the HLA *DRB1*, *DQA1* and *DQB1* allelic groups and rs2476601 in the *PTPN22* gene in 206 patients diagnosed with GD. According to our data, the proportions of the risk variant HLA-DQA1*05 as well as the risk HLA haplotype DRB1*03-DQA1*05-DQB1*02 were significantly higher in GD patients than in the general European population as given in the Allele Frequencies website. Although the tested genes individually or in combination cannot be considered reliable relapse predictors in the Czech population, the HLA-DQA1*05 allelic group showed a statistically significant association with relapse or long-term unsuccessful conservative treatment. Therefore, this variant may be a useful prognostic marker in GD patients with a difficult interpretation of their clinical picture, when treatment strategies remain unclear.

## Data Availability Statement

The datasets presented in this study can be found in online repositories. The names of the repository/repositories and accession number(s) can be found below: NCBI SRA Bio Project, accession no: PRJNA776790.

## Ethics Statement

The studies involving human participants were reviewed and approved by Ethics Committee of the Institute of Endocrinology. The patients/participants provided their written informed consent to participate in this study.

## Author Contributions

Conceptualization and design of the work, formal analysis, project administration, original draft preparation and writing, DV. Methodology of genetic analyzes, JVc and EV. Review & editing, statistics, MV. Data curation, critical revision of the work, KZ, JVr, MD, PP, and ZN. Supervision, BB. All authors contributed to the article and approved the submitted version.

## Funding

The study was supported by Ministry of Health of the Czech Republic, grant RVO 00023761.

## Conflict of Interest

The authors declare that the research was conducted in the absence of any commercial or financial relationships that could be construed as a potential conflict of interest.

## Publisher’s Note

All claims expressed in this article are solely those of the authors and do not necessarily represent those of their affiliated organizations, or those of the publisher, the editors and the reviewers. Any product that may be evaluated in this article, or claim that may be made by its manufacturer, is not guaranteed or endorsed by the publisher.
